# The Age-Friendly University Umbrella: New Synergies for Gerontology Programs, Aging Centers, and Health Systems

**DOI:** 10.1093/geroni/igaa057.1792

**Published:** 2020-12-16

**Authors:** John Schumacher

**Affiliations:** University of Maryland, Baltimore County (UMBC), Baltimore, Maryland, United States

## Abstract

Higher education has a tentative relationship with gerontology programs and research centers. Despite demographic shifts, academic gerontology serves a modest student body and attracts limited research/scholarly interest. Our new Age Friendly University (AFU) designation served to motivate our university leaders and faculty to re-imagine how aging issues are fundamental to our mission framed by: 1) Interdisciplinarity; 2) Intergenerational relations; and 3) Inclusiveness of the life course. Additionally, the AFU initiative energized the relationships among our health professions schools, academic departments, and research center to collaborate on grant opportunities. Our AFU also started collaborating with a local health system seeking Age Friendly Health System status. Finally, our AFU designation raised visibility with our three local county governments who had just received Age Friendly Community status. Our AFU efforts have served as an inclusive umbrella to attract new, interested partners in our shared effort to improve the lives of older adults. Part of a symposium sponsored by Directors of Aging Centers Interest Group.

